# Longitudinal study of human polyomaviruses viruria in kidney transplant recipients

**DOI:** 10.1007/s10238-023-01290-z

**Published:** 2024-01-17

**Authors:** Maria Dolci, Caterina Colico, Federico Ambrogi, Evaldo Favi, Lucia Signorini, Marta Perego, Edoardo Campioli, Kevin Kamau Maina, Pasquale Ferrante, Mariano Ferraresso, Serena Delbue

**Affiliations:** 1 Laboratory of Molecular Virology, Department of Biomedical, Surgical and Dental Sciences, University of Milano, Via Carlo Pascal 36, 20133 Milan, Italy; 2 Department of Emergency Surgery, Fondazione IRCCS Ca’ Granda Ospedale Maggiore Policlinico, Milan, Italy; 3 Department of Clinical Sciences and Community Health, University of Milan, Milan, Italy; 4 IRCCS Policlinico San Donato, San Donato Milanese, Milan, Italy; 5 General Surgery and Kidney Transplantation, Fondazione IRCCS Ca’ Granda Ospedale Maggiore Policlinico, Milan, Italy

**Keywords:** Immunosuppression, Kidney transplantation, Viruria, JCPyV and BKPyV replication

## Abstract

**Introduction:**

Immunosuppression after kidney transplantation (KTx) exposes recipients to *Human Polyomaviruses* (HPyVs) infections, whose natural history is still misunderstood.

**Methods:**

Allograft biopsies, and urine from 58 donor-recipient pairs were collected before KTx (T0) and 1 (T1), 15 (T2), 30 (T3), 60 (T4), 90 (T5), 180 (T6), 270 (T7), 360 (T8), and 540 (T9) days after transplant. Specimens were tested for JC (JCPyV) and BK (BKPyV), by quantitative Real-Time PCR. The course of post-KTx HPyVs viruria, and the association between JCPyV viruria in recipients and donors, were evaluated.

**Results:**

HPyVs were detected in 3/58 (5.2%) allograft biopsies. HPyVs viruria was present in 29/58 (50%) donors and 41/58 (70.7%) recipients. JCPyV DNA was detected in 26/58 (44.8%) donors and 25/58 recipients (43.1%), 19 of whom received kidney from JCPyV positive donor, whereas BKPyV genome was detected in 3 (5.2%) donors and 22 (37.9%) recipients. The median time of JCPyV, and BKPyV first episode of replication was 1, and 171 days post KTx, respectively. At T0, JCPyV viruria of donors was associated with increased risk of JCPyV replication post-KTx; recipients with JCPyV positive donors showed lower risk of BKPyV replication post-KTx.

**Conclusions:**

The results suggested that JCPyV may be transmitted by allograft, and that its replication post KTx might prevent BKPyV reactivation. Future investigation regarding correlation between chronic exposure to immunosuppressive agents and HPyVs urinary replication are warranted.

**Supplementary Information:**

The online version contains supplementary material available at 10.1007/s10238-023-01290-z.

## Introduction

*Human Polyomaviruses* (HPyVs) are small, non-enveloped DNA viruses [1]. HPyVs family includes 14 polyomaviruses [2] with JC Polyomavirus (JCPyV) and BK Polyomaviruses (BKPyV) representing the most extensively investigated ones. The prevalence of HPyVs infection in the general population is very high, with seroprevalence rates ranging from 60 to 100%, depending on the series [3]. It is known that HPyVs can establish latency in kidney tubular epithelial cells [4–6]. Transient or chronic immunosuppression are risk factors for viral reactivation [7]. JCPyV is the causative agent of progressive multifocal leukoencephalopathy (PML)[8], and BKPyV is etiological agent of Polyomavirus-associated Nephropathy (PVAN) [9]. Anti-rejection prophylaxis after kidney transplantation (KTx) is considered as the main trigger of latent HPyVs reactivation [10], but all these viruses may also be transmitted through the allograft [11–14]. Polyomavirus-induced diseases can increase morbidity and mortality in solid organ transplant recipient [15]. In KTx, BKPyV nephropathy, occurring in 1–10% of KTx [16], are associated with renal dysfunction and premature allograft loss, and, to date, no therapeutical strategies are still approved. Whilst the role of BKPyV and PVAN in KTx is well recognized, the link between JCPyV infections and renal allograft dysfunction remains unclear.

The aim of this prospective observational study with 18 months of follow-up was to assess HPyVs prevalence and replication pattern in renal biopsies and urine samples of a cohort of consecutive KTx donor-recipient pairs. Risk factors for post-transplant HPyVs infection or reactivation were also investigated, particularly focusing on donor and recipient HPyVs-specific immunization and recipient immunosuppression.

## Materials and methods

### Study design

**Fig. 1:**
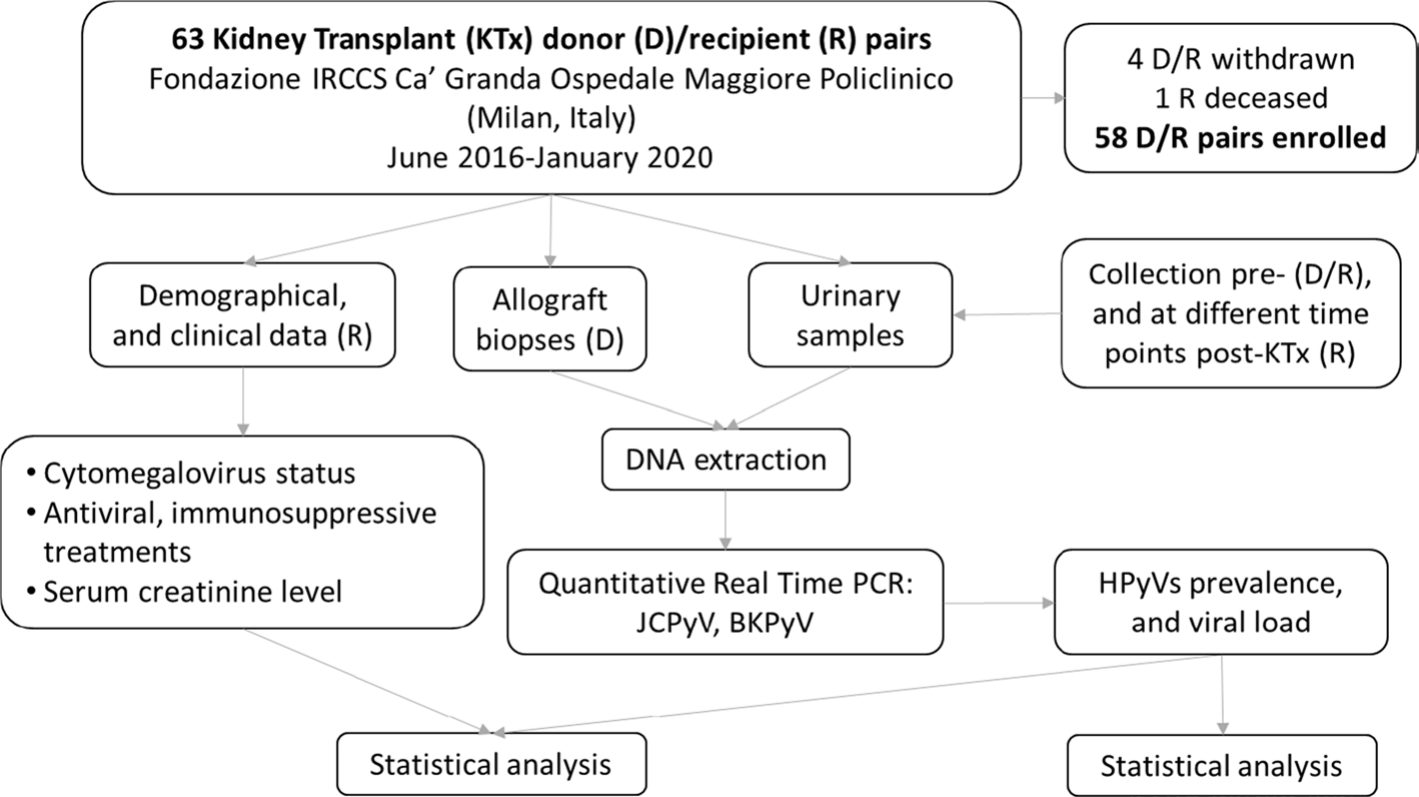
Experimental overview. Between June 2016 and January 2020, fifty-eight kidney transplant recipients (R) and as many donors (D) were enrolled. For each patient (R), demographical, and clinical data, allograft biopsies (D) and urinary samples (D/R) were collected. By means of Real Time PCR, HPyVs prevalence and viral load were evaluated. Association between clinical data and HPyVs positivity was also investigated by statistical analysis

A single-centre prospective observational study was performed to investigate the origin and pattern of HPyVs replication in KT recipients [17]. There were no exclusion criteria. Eventually, the study population included 63 donor-recipient pairs (a flow diagram of the study is depicted in Fig. 1) but 5 patients were withdrawn and one patient died. So, a total of 58 donor-recipient pairs were enrolled. Donor-related procedures were executed in hospital under the area of influence of the Nord Italia Transplant (NITp) Organization. All KT procedures were carried out at the Fondazione IRCCS Ca’ Granda Ospedale Maggiore Policlinico (Milan, Italy) between June 2016 and January 2020. The study was approved by the Fondazione IRCCS Ca’ Granda Ospedale Maggiore Policlinico Ethical Committee (protocol 2191) and conducted according to the World Health Organization Declaration of Helsinki and applicable regulatory requirements. Participating subjects or their legal representatives signed a specific informed consent. Demographic characteristics (age, gender), type of donation (living/deceased donor), primary renal disease, time on dialysis before transplant, number of HLA mismatches, cold ischemia time, induction and maintenance immunosuppression were collected at baseline.

### Specimens processing and DNA isolation

During bench preparation of the kidney allograft, a wedge tissue biopsy from kidney cortex was taken and stored in RNA later solution (Qiagen, Hilden, Germany) at − 80 °C. Total urine samples were collected from the donor and the recipient at the time of organ retrieval and immediately before transplant, respectively (T0); the specimens were promptly stored at − 80 °C in a dedicated laboratory facility. Further urinary specimens were collected from the recipient at 1 (T1), 15 (T2), 30 (T3), 60 (T4), 90 (T5), 180 (T6), 270 (T7), 360 (T8), and 540 (T9) days after transplant.

Allograft biopsies were assessed for JCPyV and BKPyV genome, using the combination of QIazol and QIAmp DNA Mini and Blood Mini kit (Qiagen, Hilden, Germany), according to manufacturer’s protocols. Donor and recipient urine samples were assessed for viral genomes using the NucleoSpin RNA virus kit (Macherey–Nagel, Allentown, PA).

### HPyVs load quantification in renal biopsies and urine samples

All specimens were evaluated for the presence of HPyVs genomes by quantitative Real-Time PCR using the 7500 Applied Biosystem (Thermo Fisher Scientific, Waltham, MA). A singleplex for JCPyV and for BKPyV DNA were performed. The sequences of the primers and probes as well as the composition of the reaction mix and the thermal cycle of the Real-Time PCR have already been reported in previous publications [18, 19]. Viral loads in urine samples and renal biopsies were expressed as copies/mL and copies/µg, respectively.

### Immunosuppressive protocols and concomitant medications

As induction, low-immunological risk patients (first transplant, last panel reactive antibody test < 50%, undetectable donor specific antibody, donor-recipient HLA mismatch < 5, standard criteria donor) received intravenous (IV) basiliximab (Simulect®, Novartis, Basel, Switzerland) 20 mg on day 0 and day 4 whereas those with high-immunological risk received IV rabbit anti-thymocyte globulin (rATG, Thymoglobulin®, Genzyme, Cambridge, MA) 5 mg/kg total-dose from day 0 to day 4. All recipients were also given IV methylprednisolone 250 mg on day 0, 125 mg on day 1 and day 2.

As maintenance, all patients were given a triple-agent immunosuppressive scheme including orally administered standard-release (Adoport®, Novartis International, Basel, Switzerland) or extended-release (Advagraf®, Astellas Pharma, Tokyo, Japan) tacrolimus, mycophenolate mofetil (MMF, Myfenax®, Teva, Petach Tikva, Israel) or mycophenolic acid (MPA, Myfortic®, Novartis International, Basel, Switzerland), and prednisone. Standard-release and extended-release tacrolimus were started on day 0 and the dose was adjusted to achieve a trough level of 8–12 ng/mL during the first month and 6–8 ng/mL thereafter. MMF and MPA were administered from day 0 (basiliximab induction) or day 5 (rATG induction) using 1000 mg or 720 mg twice daily, respectively. From day 3, patients received oral prednisone 20 mg/day, progressively tapered to 5 mg/day after 1 month.

All recipients were given oral prophylaxis for Pneumocystis jirovecii (trimethoprim/sulfamethoxazole 80 + 400 mg/day three times a week for three months). Patients at increased risk of *Cytomegalovirus* (CMV) disease (recipient with negative CMV immunization receiving a kidney from a donor with positive CMV immunization and/or treated with rATG) were given oral valganciclovir (dose titrated according to renal function) for three to six months; other recipients were managed according to a pre-emptive strategy.

### Other data collection

The graft ureter was anastomosed to the bladder using two 6/0 polydioxanone running sutures over a double J ureteral stent, according to the Lich-Gregoir technique. The stent was removed under cystoscopy between 4 to 6 weeks after surgery.

Primary non-function (PNF) was defined as graft function unable to prevent continued renal replacement therapy or requiring re-transplantation when surgical causes of transplant failure or established medical complications were excluded (by imaging, exploration and/or histology). Delayed graft function (DGF) was defined as the need for dialysis during the first week after transplant. Diagnosis of rejection was based on serum creatinine concentration increase ≥ 30% from nadir and confirmed by histology whenever possible [20]. Renal function was measured by serum creatinine concentration (SCr, mg/dL) and estimated glomerular filtration rate (eGFR, mL/min/1.73 m2) using the Modification of Diet in Renal Disease (MDRD) formula [21]. As per local practice, all transplant recipients were also screened for BKPyV viremia by plasma quantitative PCR. The test was performed monthly during the first six months of follow-up and in case of worsening graft function (SCr increase ≥ 30% from nadir) thereafter. Patients with BKPyV plasma quantitative PCR ≥ 1000 copies/mL were diagnosed as BKPyV viremic. Whenever feasible, those with BKPyV quantitative PCR ≥ 10,000 copies/mL were further assessed by allograft histology (ultrasound-guided biopsy) to rule out PVAN [22].

Recipients with persistent (≥ four weeks) BKPyV viremia, regardless of symptoms or histology, had their immunosuppressive therapy progressively modified according to the following scheme: (1) 50% reduction of MMF or MPA; (2) withdrawal of MMF or MPA; (3) 30% reduction of standard-release or extended-release tacrolimus; (4) 50% reduction of standard-release or extended-release tacrolimus; and (5) switch from tacrolimus to cyclosporine (Neoral®, Novartis International; Basel, Switzerland). Sequential changes were made every two weeks according to clinical findings. Response to treatment was defined as: (a) Complete (no viremia with restored graft function); (b) partial (no viremia with permanently impaired graft function); and (c) absent (persistent viremia with progressive graft failure due to PVAN) [22].

### Statistical analysis

The distributions of HPyVs replication were described with proportions or percentages and compared using the Chi-Square test. HPyVs loads were described with means (± standard deviation) and compared using the Student’s T-test. Correlations with *p-value* < 0.05 were considered as statistically significant.

The non-parametric Kaplan–Meier estimator was used to evaluate the probability of post-transplant HPyVs replication according to specific donor or recipient characteristics. The Cox proportional hazard model was used to evaluate the association through hazard ratios. A Cox regression model with a time-dependent variable (the infectious status developed during follow-up), was used to evaluate the association between the risk of infection of one virus in relation to the patient infectious status of other viruses during follow-up.

Analyses were performed using SPSS (version 23.0; IBM Corp., Armonk, NY, USA).

## Results

### Demographic and clinical characteristics of the study population

Donor, recipient, and transplant-related characteristics are detailed in Table 1, while transplant-related outcomes after 18 months of follow-up are summarized in Table 2.

**Table 1:** Characteristic of the study population (n = 58)

Donor variables	n (%) or mean / median value
Male: Female	28 (48.3): 26 (44.8)
	4 not available
Ethnicity	Italian 54 (93.1)
	Albanian 1 (1.7)
	Colombian 1 (1.7)
	Romanian 1 (1.7)
	Sri Lankan 1 (1.7)
Donor source, Living: Deceased	33 (56.9%): 25 (43.1%)
Age (years)	54.4 ± 14.4 years (range: 79.5–8.3 years)
Recipient variables	n (%) or mean / median value
Male: Female	31 (53.5): 27 (46.6)
Ethnicity	Italian 51 (87.9)
	Philippine 2 (3.5)
	Austrian 1 (1.7)
	Ethiopian 1 (1.7)
	Kenyan 1 (1.7)
	Lebanese 1 (1.7)
	Tunisian 1 (1.7)
Age at transplant (years)	42.2 ± 19.0 years (range: 74.1–4.4 years)
*Primary disease*	
- Autosomal Dominant Polycystic Kidney Disease (ADPKD)	11 (19.0)
- Focal segmental glomerulosclerosis (FSGS)	4 (6.9)
- IgA nephropathy	4 (6.9)
- Systemic lupus erythematosus (SLE)	3 (5.2)
- Vesicoureteral reflux (VUR)	3 (5.2)
- Atypical Hemolytic Uremic Syndrome	2 (3.5)
- Alport syndorme	2 (3.5)
- Goodpasture syndrome	2 (3.5)
- Other	18 (31.0)
- Not determined	9 (15.5)
Pre-emptive transplant	14 (24.1)
Average Time on dialysis before transplant (months)	21 (range: 110–0)
HLA mismatches A-B-DR-DQ (n)	
0—2	14 (24.1)
3—4	20 (34.5)
5—6	20 (34.5)
nd	4 (6.9)
Mean Cold ischemia time (hours)	7:28 (range: 17:15–00:02)
*Induction agent*	
- Basiliximab	35 (60.3)
- Anti-thymocyte globulin	22 (37.9)
- Methylprednisolone	57 (98.3)
*Maintenance agent:*	
- Standard or extended-release tacrolimus	57 (98.3)
- MMF or MPA	57 (98.3)
- Prednisone	57 (98.3)
CMV prophylaxis: Pre-emptive strategy	29 (50.0)

**Table 2:** Transplant outcomes

Variables	n (%) or mean / median value
1 Y Patient survival (%)	58 (100.0)
1 Y Death-censored allograft survival (%)	56 (96.6)
Primary non-function (PNF) (%)	0
Delayed Graft Function (DGF) (%)	9 (15.5)
1 Y Rejection rate:	
- Cell-mediated	1 (1.7)
- Antibody-mediated	0
1 Y CMV disease (%)	10 (17.2)
Allograft function (SCr / GFR):	
0–15 d	1.4 ± 0.8
15–30 d	1.3 ± 0.5
30–60 d	1.5 ± 0.6
60–90 d	1.4 ± 0.7
90–180 d	1.4 ± 0.8
180–270 d	1.3 ± 0.3
270–360 d	1.4 ± 0.9
360–540 d	1.5 ± 1.5
> 540 d	1.3 ± 0.3

### HPyVs prevalence and load in pre-transplant allograft biopsies and in urinary samples of donors

Overall, HPyV genomes were detected in 3/58 (5.2%) pre-transplant allograft biopsies (Table 3). More in details, two (3.4%) specimens were positive for JCPyV (viral load: 1.0E + 01 and 2.5E + 01 copies/µg), and one (1.7%) for BKPyV (viral load: 1.8E + 04 copies/µg).

**Table 3:** HPyVs prevalence and load in biopsies and in urine samples of the donors

	JCPyV		BKPyV	
Sample	Positive/Total (%)	Median viral load [range]	Positive/Total (%)	Median viral load [range]
Biopsy	2/58 (3.4%)	1.75E + 01 [1.0E + 01—2.5E + 01] copies/ug	1/58 (1.7%)	1.8E + 04 copies/ug
Urine	26/58 (44.8%) ^#^	7.8E + 05 [1.2E-02–2.2E + 08] copies/mL	3/58 ^#^ (5.2%)	1.8E + 03 [5.7E + 02 – 3.7E + 03] copies/mL

^#^ p < 0.0001

HPyVs were detected in the urinary samples collected from 29/58 (50.0%) donors (Table 3). Precisely, JCPyV viruria was recorded in 26/58 (44.8%) patients (median viral load: 7.8E + 05 copies/mL, range: 1.2E + 02—2.2E + 08 copies/mL), whereas BKPyV viruria in 3/58 (5.2%) subjects (median viral load: 1.8E + 03 copies/mL, range: 5.7E + 02—3.7E + 03 copies/mL).

We observed that the proportion of donors with JCPyV viruria was significantly higher than that with BKPyV viruria (*p* < 0.0001). On the contrary, JCPyV and BKPyV loads in urinary samples were not significantly different.

### HPyVs prevalence and load in urinary samples of recipients

Overall, HPyVs viruria was recorded in 41/58 (70.7%) recipients (Table 4). JCPyV was detected in the urinary samples collected from 25/58 (43.1%) subjects (median viral load: 1.3E + 06 copies/mL, range: 1.0E + 02—1.6E + 10 copies/mL). Among these patients, 19/25 (76.0%) received a kidney from a JCPyV positive donor. BKPyV viruria was present in 22/58 (37.9%) recipients (median viral load: 8.7E + 04 copies/mL, range: 4.7E + 01—6.7E + 09 copies/mL).

**Table 4:** HPyVs prevalence and load in urinary samples of recipients

	JCPyV	BKPyV
Positive/Total(%)	25/58^#^ (43.1%)	22/58^#^ (37.9%)
Median viral load [range] copies/mL	8.73E + 04 [1.1E + 02–1.6E + 10]	1.36E + 01 [4.7E + 01–6.7E + 09]

^#^ 6 recipient subjects co-infected with JCPyV and BKPyV

Simultaneous JCPyV plus BKPyV viruria was detected in six subjects. HPyVs prevalence and urinary loads were not significantly different.

### Course of post-transplant JCPyV and BKPyV viruria

The proportion of recipients with JCPyV viruria at baseline (T0) was 2/58 (3.5%). As shown in Table 5, we observed a progressive and consistent increase in the number of patients with JCPyV urinary replication over time, reaching a peak at T8 (23/58, 39.7%).

**Table 5:** Time-dependent distribution of JCPyV and BKPyV viruria after transplant

Time after transplant	JCPyV + /tot	% JCPyV +	BKPyV + /tot	% BKPyV +
T0	2/58	3.5	2/58	3.5
T1	14/58	24.1	2/58	3.5
T2	14/58	24.1	1/58	1.7
T3	16/58	27.6	3/58	5.2
T4	17/58	29.3	6/58	10.3
T5	16/58	27.6	7/58	12.1
T6	19/58	32.8	9/58	15.5
T7	21/58	36.2	7/58	12.1
T8	23/58	39.7	9/58	15.5
T9	21/58	36.2	15/58	25.9

As for BKPyV, the proportion of recipients with viruria at baseline (T0) was 2/58 (3.5%): there was an increase in the number of patients exhibiting BKPyV urinary replication over time with a peak at T9 (15/58, 25.9%) (Table 5).

The median time from transplant to first episode of JCPyV viruria was one day.

The time-dependent distribution of the first episodes of JCPyV viruria was also evaluated. We found that the number of patients with new-onset viral replication in the urine was two (2/25, 8.0%) at T0, 14 (14/25, 56.0%) at T1, two (2/25, 8.0%) at T2, three (3/25, 12.0%) at T6, one (1/25, 4.0%) at T7, two (2/25, 8.0%) at T8, and one (1/25, 4.0%) at T9.

The median time from transplant to first episode of BKPyV viruria was 171 days.

The number of patients with new-onset viral replication in the urine was two (2/12, 16.7%) at T0, one (1/22, 4.5%) at T1 and T2, two (2/22, 9.1%) at T3, T4, and T5, four (4/22, 18.2%) at T6, one (1/22, 4.5%) at T7, two (2/22, 9.1%) at T8, and five (5/22, 22.7%) at T9.

**Fig. 2:**
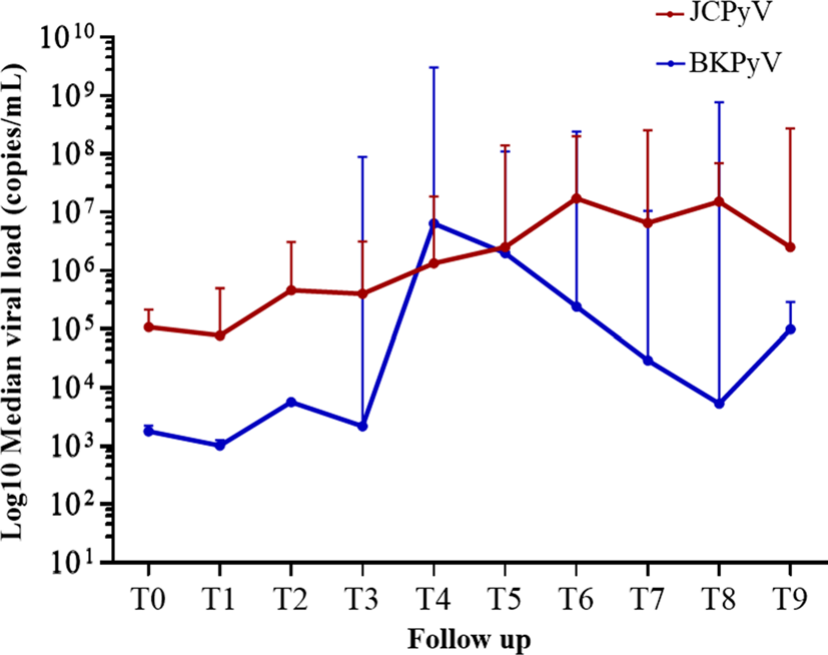
Post-transplant JCPyV (red line) and BKPyV (blue line) urinary load (median, 1st—3rd interquartile). Follow-up specimens were collected before KTx (T0), and at 1 (T1), 15 (T2), 30 (T3), 60 (T4), 90 (T5), 180 (T6), 270 (T7), 360 (T8), 540 (T9) days after transplantation

Median JCPyV and BKPyV urinary load at every time point is summarized in Fig. 2 and Tables S1 and S2.

### Association between JCPyV replication in recipients and HPyVs replication in donors

**Fig. 3:**
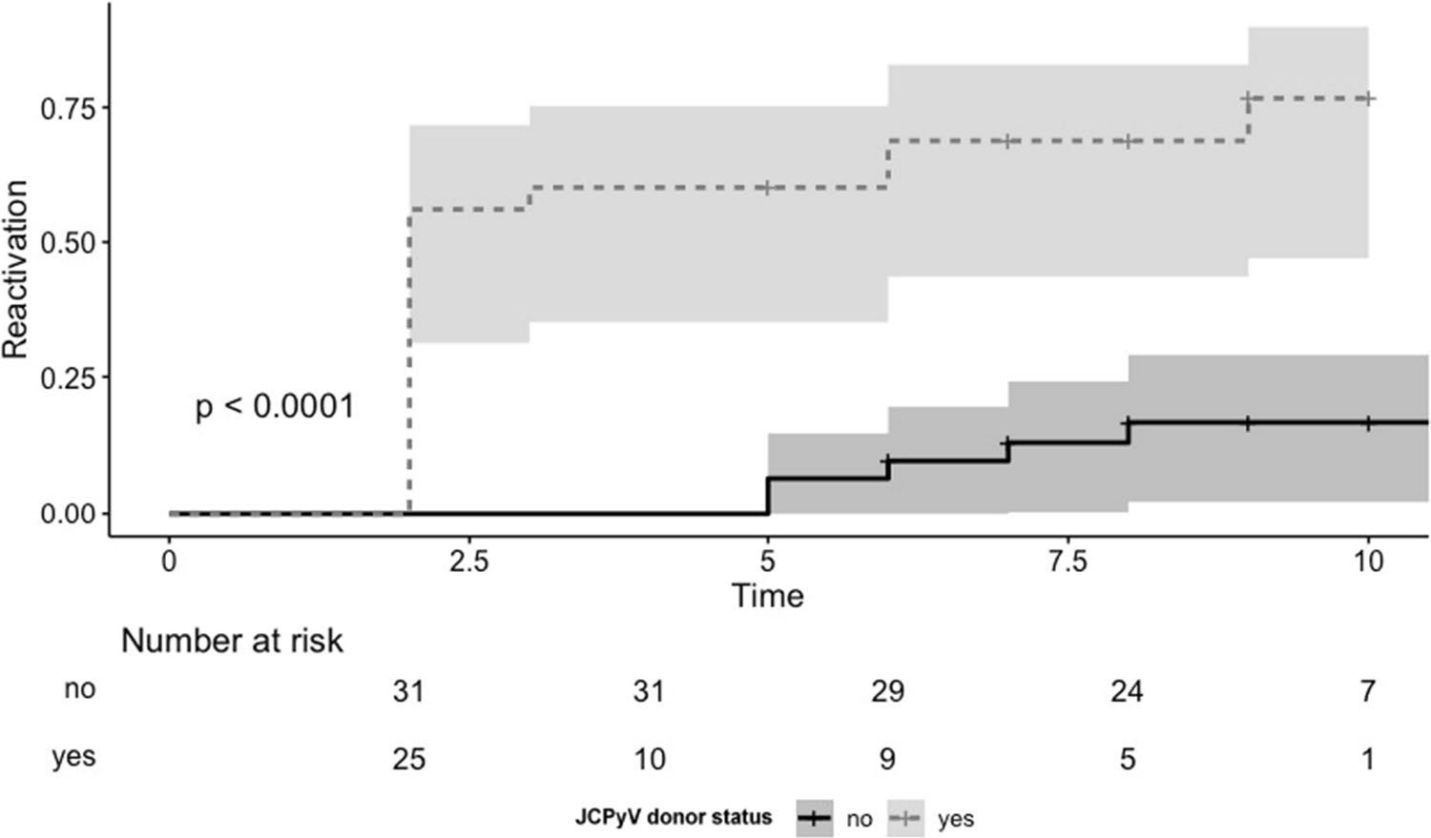
Post-transplant JCPyV viruria according to donor JCPyV viruria

As depicted in Fig. 3, the prevalence of donor JCPyV viruria at the time of organ procurement was associated with a significant increase in the risk of post-transplant JCPyV urinary replication (JCPyV positive vs. negative donor: 8.5; 95%CI 3.1–23.7; *p* < 0.0001). On the contrary, JCPyV donor status did not influence the JCPyV load in the urine of recipients (JCPyV mean load in recipients’ urine with negative donor: 5.9E + 06 copies/mL vs JCPyV mean load in recipients’ urine with negative donor: 5.7E + 08 copies/mL).

BKPyV (BKPyV positive vs. negative donor: 0.4; 95%CI 0.1–3.1; *p* = 0.40) urinary replication in the donor did not show any relevant effect on the likelihood of JCPyV viruria in the recipients.

Also, no associations were detected between post-transplant JCPyV viruria and BKPyV viruria in recipients (BKPyV positive vs. negative recipient: 0.6; 95%CI 0.1–3.0; *P* = 0.53).

### Association between BKPyV replication in recipients and HPyVs replication in donors

**Fig. 4:**
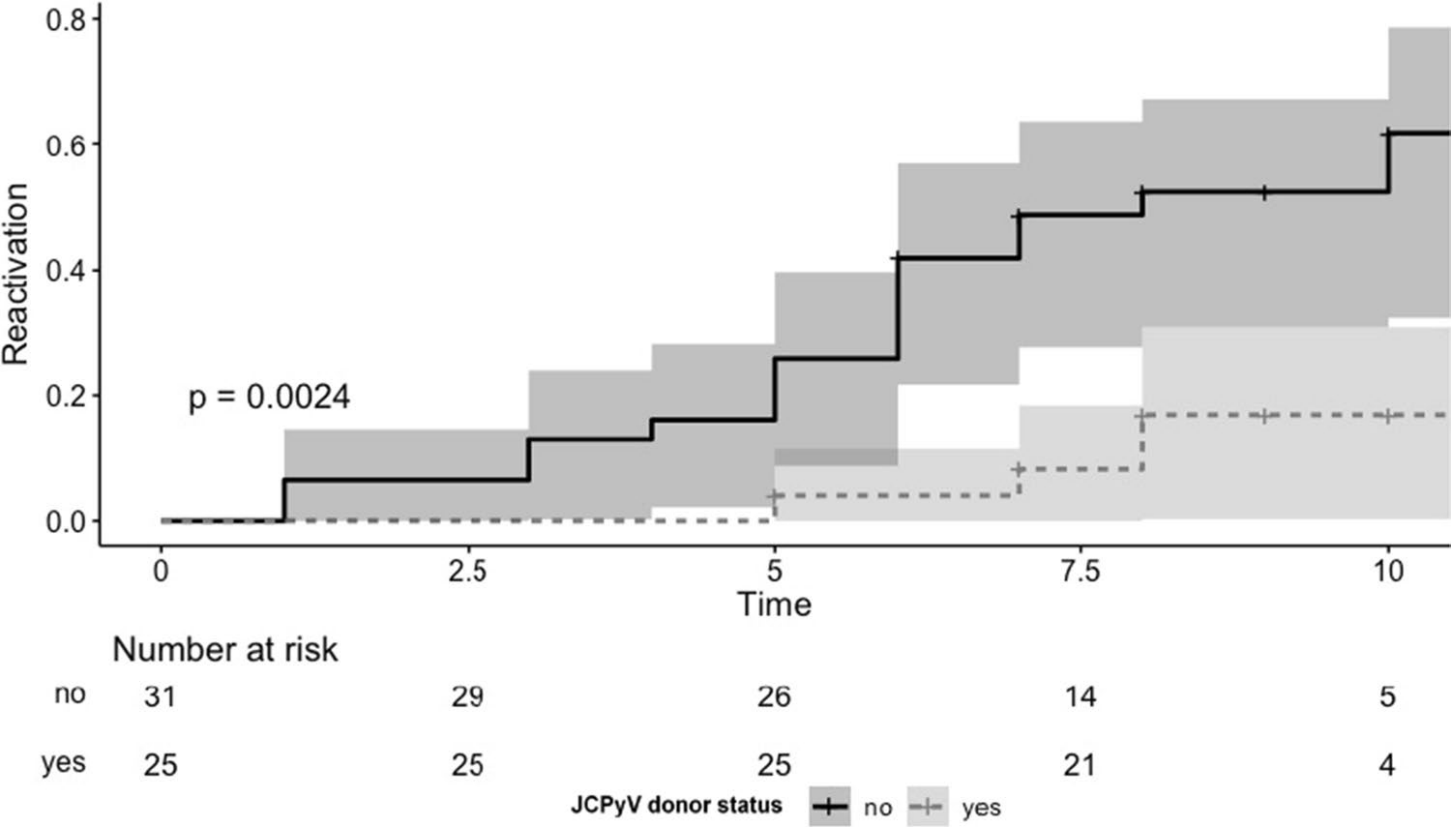
Post-transplant BKPyV viruria according to donor JCPyV viruria

As depicted in Fig. 4, patients receiving a kidney from donor with JCPyV viruria showed a significantly lower risk of post-transplant BKPyV urinary replication than those transplanted from a JCPyV negative donor (JCPyV positive vs. negative donor: 0.2; 95%CI 0.1–0.6; *p* = 0.006). However, JCPyV donor status did not influence the BKPyV load in the urine of recipients (BKPyV mean load in recipients’ urine with negative donor: 9.9E + 06 copies/mL vs BKPyV mean load in recipients’ urine with negative donor: 1.8E + 04 copies/mL).

We could not find any relationship between BKPyV viruria of the donor (BKPyV positive vs. negative donor: 1.4; 95%CI 0.3–5.9; *p* = 0.67) and post-transplant BKPyV urinary replication.

During follow-up, we observed that recipients with JCPyV viruria exhibited a reduced risk of post-transplant BKPyV viruria than those with no signs of JCPyV urinary replication (JCPyV positive vs. negative recipient: 0.2; 95%CI 0.04–0.7; *p* = 0.02).

### Association between HPyVs viruria of recipients and allograft function

We could not find any significant association between JCPyV or BKPyV urinary replication in recipients and post-transplant SCr (*P* = 0.59 for JCPyV, and *P* = 0.46 for BKPyV).

### Association between anti-rejection medications or CMV prophylaxis and post-transplant HPvVs viruria

**Fig. 5:**
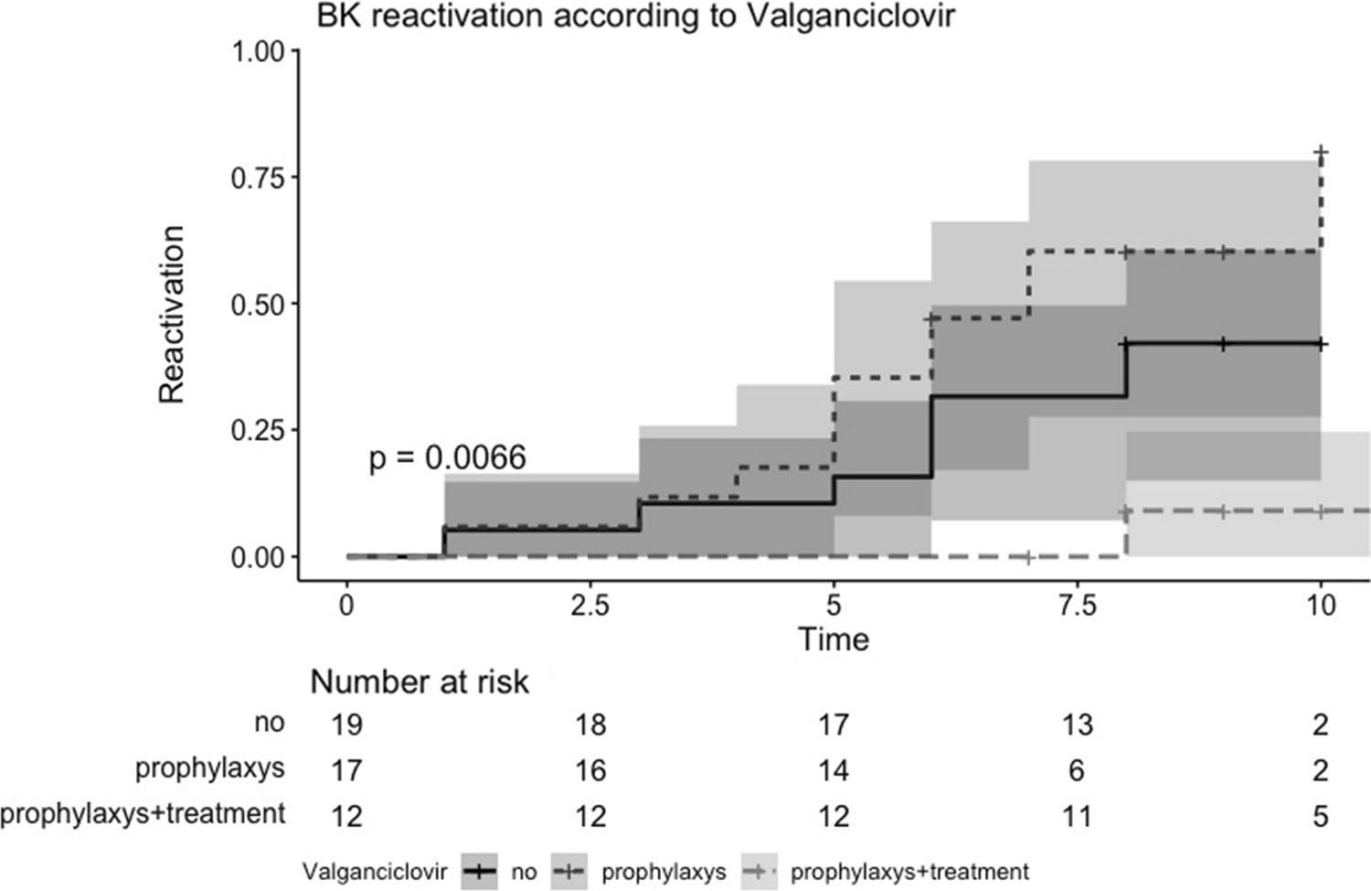
Relationship between post-transplant BKPyV urinary replication and valganciclovir administration

As shown in Table S4, there were no significant associations between the administration of specific induction (basiliximab vs. rATG) or maintenance (tacrolimus vs. MMF/MPA vs. steroid) immunosuppressive drugs and post-transplant JCPyV or BKPyV urinary replication. On the contrary, there was an inverse relationship between valganciclovir administration and post-transplant BKPyV viruria (Fig. 5). Precisely, we observed that BKPyV urinary replication was significantly less frequent among patients who had received universal prophylaxis or pre-emptive therapy than controls (*p* = 0.0066).

## Discussion

KTx is the best therapeutic option for ESRD. Nevertheless, the need for chronic immunosuppression exposes transplant recipients to serious complications such as drug-related toxicity, cardiovascular disease, infections, and malignancy [23–26]. Among the others, HPyV infections represent as a major cause of post-transplant morbidity and premature allograft loss [27, 28].

Despite recent advances in the understanding of HPyVs epidemiology and biology [17, 22], the origin and the natural history of BKPyV and JCPyV infections in KTx recipients have not been clarified. In particular, there is a lack of information regarding HPyVs behavior and reciprocal interactions in asymptomatic patients. In the present study, we have prospectively assessed JCPyV and BKPyV urinary replication in a cohort of KT recipients. Viruria of donors and pre-transplant intra-allograft replication were also evaluated.

We found that JCPyV viruria was particularly frequent, in both donors and recipients. Moreover, we observed that the proportion of donors and recipients with JCPyV urinary replication was significantly higher than BKPyV. These results are overall in line with previously published data [29–31]. However, some authors have reported an higher prevalence of BKPyV viruria, particularly among recipients [32–35]. Such discrepancy may be due to several reasons including different geographical or ethnic characteristics or it may reflect the fact that BKPyV is more frequently associated with clinical manifestations than JCPyV. In this regard, it is worth mentioning that, in our series, samples collection was not clinically-driven, but preliminarily planned as a part of the study follow-up.

As reported by other groups, JCPyV urinary replication was significantly more frequent in patients receiving a kidney from a donor with JCPyV viruria at the time of organ procurement [11, 12, 36], 13]. In a recent study, we have demonstrated that there is a very strong concordance between donor and recipient JCPyV genotypes [17]. This observation suggests that peri-transplant recipient JCPyV viruria is mostly secondary to a direct virus transmission from the donor to the recipient via the allograft [17]. The fact that the median time from transplant to first JCPyV urinary excretion was only about one day certainly supports this hypothesis [34, 36, 37].

The pattern of JCPyV urinary replication during the post-transplant follow-up was similar to those described in other prospective studies on KT recipients or healthy subjects and overall suggests that the virus can remain latent in renal tubular epithelial cells, easily reactivating in case of transient or persistent states of immunosuppression [32, 35, 38–41].

Given the small sample size and the short follow-up, no episodes of PML were recorded. Also, no correlations between JCPyV viruria and allograft function or other adverse outcomes could be detected. Therefore, our experience further confirms that there is no need for systematic JCPyV screening before and after transplant.

The prevalence of BKPyV viruria among our recipients was lower than expected [34]. Furthermore, the median time from transplant surgery to the first recorded episode of viruria was longer than those described by previously published studies [37, 42], but in line with the one recently reported by Querido and colleagues [35]. The prevalence of BKPyV viruria and BKPyV urinary load were highest around T4, with a steady decrease during the rest of the follow-up. For instance, no cases of BKPyV-related allograft dysfunction were recorded. Rejection rate was also extremely low. These data seem to suggest that BKPyV urinary replication is not primarily determined by induction or early maintenance immunosuppressive therapies, but more likely by the chronic burden of immunosuppression. The difference between our results and other reports is difficult to explain [34]. The fact that our population included many living-donor and low-immunological risk recipients (requiring a lower degree of immunosuppression) may have played a role. As a matter of fact, our experience confirms that early screening for BKPyV infection may not be appropriate in standard-risk KT [22].

Simultaneous BKPyV viruria in both donor and recipient was quite uncommon compared to JCPyV. Certainly, the paucity of events recorded prevents any meaningful conclusion. Nevertheless, we have already shown with sequencing analysis that, in this studied cohort, the concordance between donor and recipient BKPyV strains was rather low, thus speculating that at least some episode of post-transplant BKPyV replication is likely due to reactivations of latent viruses in the recipients, while some other reactivation episodes might be due to the donor transmission [17].

Analyzing the distributions and the entities of JCPyV and BKPyV urinary replications in donors and recipients, we observed that the subjects with JCPyV viruria were less prone to develop BKPyV viruria. As proposed by Saundh et al. or Cheng and colleagues, this finding suggests that JCPyV may interfere with BKPyV replication [34]. The possible mechanisms underlying this phenomenon are undetermined. The high genomic similarity of the antigenic proteins of JCPyV and BKPyV could promote a sort of immune cross protection [43–45] or, more likely, JCPyV could compete for the very same cellular replication machinery as BKPyV [34]. Future studies should include baseline serological evaluation of donors and recipients, and possibly focus on this interesting interaction.

Our study was clearly underpowered to detect any significant association between baseline immunosuppression and the risk of post-transplant HPyVs viruria. However, as described in larger populations, the prevalence of JCPyV and BKPyV viruria in patients receiving basiliximab or rATG was similar [22, 46]. Remarkably, we found that recipients on CMV universal prophylaxis were less likely to exhibit HPyVs viruria than controls. Although we cannot provide any matching information regarding HPyVs and CMV replication in blood or urine, these data confirm a possible relationship between CMV and BKPyV infection [22, 47].

The impact of transient or prolonged HPyVs viruria on transplant-related outcomes is still controversial. As previously discussed, we did not observe any episode of PML or PVAN. Prospective assessment of HPyVs viruria and SCr also failed to demonstrate a possible effect of HPyVs urinary replication on allograft function [48–50].

We recognize that our study has several limitations including a relatively small sample size, the lack of information regarding baseline HPyVs serological status, no systematic screening for post-transplant HPyVs viraemia, and no follow-up allograft histology. Nevertheless, our findings will serve as a basis for future research investigating the correlation between chronic exposure to specific immunosuppressive agents and HPyVs urinary replication. Due to the lack of approved antiviral therapies, if our results will be confirmed, the late screening for BKPyV infection after KT may improve the clinical practice impact in these patients.

## Supplementary Information

Below is the link to the electronic supplementary material.

Supplementary file1 (DOCX 17 kb)

## Data Availability

The data that support the findings of this study are available from the corresponding author upon reasonable request.
