# Supplementary material for: Longitudinal study of human polyomaviruses viruria in kidney transplant recipients
Source: Clin Exp Med. 2024 Jan 17;24(1):3. doi: 10.1007/s10238-023-01290-z (PMC10794280; doi:10.1007/s10238-023-01290-z)
Supplement: Supplementary file 1 — Supplementary file1 (DOCX 17 kb) [file 10238_2023_1290_MOESM1_ESM.docx]

|  | **T0** | **T1** | **T2** | **T3** | **T4** | **T5** | **T6** | **T7** | **T8** | **T9** |
| --- | --- | --- | --- | --- | --- | --- | --- | --- | --- | --- |
| Median | 1.04E+05 | 7.51E+04 | 4.50E+05 | 3.90E+05 | 1.30E+06 | 2.48E+06 | 1.70E+07 | 6.36E+06 | 2.45E+07 | 1.00E+07 |
| Min | 1.01E+02 | 5.42E+03 | 7.05E+02 | 2.04E+02 | 3.34E+02 | 4.58E+03 | 6.44E+02 | 1.70E+02 | 1.92E+02 | 7.50E+02 |
| Max | 2.09E+05 | 4.79E+06 | 1.80E+07 | 5.17E+07 | 1.23E+08 | 2.68E+08 | 1.08E+09 | 1.60E+10 | 5.02E+09 | 2.09E+09 |
| IQR | 1.04E+05 | 2.73E+05 | 2.21E+06 | 2.69E+06 | 1.50E+07 | 1.13E+08 | 1.59E+08 | 1.08E+08 | 1.36E+08 | 2.68E+08 |

**Table S1:** JCPyV viruria (copies/ml) at each time point for positive recipients

**Table S2:** BKPyV viruria (copies/ml) at each time point for positive recipients

|  | **T0** | **T1** | **T2** | **T3** | **T4** | **T5** | **T6** | **T7** | **T8** | **T9** |
| --- | --- | --- | --- | --- | --- | --- | --- | --- | --- | --- |
| Median | 1.83E+03 | 1.05E+03 | 5.86E+03 | 2.24E+03 | 6.56E+06 | 2.04E+06 | 2.50E+05 | 2.99E+04 | 5.50E+03 | 1.02E+05 |
| Min | 1.35E+03 | 8.00E+02 | / | 1.08E+03 | 4.42E+04 | 1.96E+04 | 1.28E+04 | 4.28E+03 | 4.70E+01 | 1.54E+03 |
| Max | 2.30E+03 | 1.30E+03 | / | 9.15E+07 | 6.73E+09 | 2.27E+08 | 8.62E+08 | 1.22E+09 | 1.58E+09 | 1.30E+06 |
| IQR | 4.75E+02 | 2.50E+02 | / | 4.57E+07 | 1.43E+09 | 6.56E+07 | 3.83E+07 | 6.10E+06 | 1.30E+06 | 2.25E+05 |

**Table S4:** Association between patients’ treatment and HPvVs viruria

| **Virus** | **Treatment** | | |
| --- | --- | --- | --- |
|  | **Valganciclovir** | **ATG** | **Basiliximab** |
| JCPyV | p = 0.35 | p = 0.34 | p = 0.22 |
| BKPyV | **p = 0.007** | p = 0.13 | p = 0.08 |
|  |  |  |  |
